# IMPACT OF MITOCHONDRIAL MALFUNCTION IN AGING IS MEDIATED BY IMPAIRED LYSOSOMES AND ENDOPLASMIC RETICULUM

**DOI:** 10.1093/geroni/igad104.1938

**Published:** 2023-12-21

**Authors:** Nuno Raimundo

**Affiliations:** Penn State College of Medicine, Hershey, Pennsylvania, United States

## Abstract

The hallmarks of aging are well established, and include mitochondrial malfunction, deregulated nutrient sensing, loss of proteostasis, and senescence, among others. While these hallmarks are all part of the aging process, they have typically been studied independently. We have found that mitochondrial malfunction can affect lysosomal activity, impacting nutrient sensing, and our that the crosstalk between mitochondria and other organelles such as endoplasmic reticulum and lysosomes affects cellular senescence, proteostasis and intercellular communication. Chronic mitochondrial malfunction causes perturbations in the lysosomal membrane which affect the function of this organelle as well as the recruitment of folliculin (FLCN) to the lysosomes. FLCN regulates the balance between anabolism (stimulating mTORC1) and catabolism (repressing AMPK). Increased recruitment of FLCN to the lysosomal membrane in cells subjected to chronic mitochondrial malfunction results in hyperactive mTORC1 and inhibition of AMPK signaling, thus aberrantly promoting anabolism under conditions that are not optimal. Furthermore, peroxisomal β-oxidation increases, and expression of proteasome subunits decreases, with consequent perturbation of proteostasis. Overall, the impact of chronic mitochondrial malfunction over lysosomes triggers multiple perturbations in organelles and organelle crosstalk that culminate in aberrant nutrient sensing, metabolic rewiring, proteostasis impairment, senescence and release of sterile inflammation-related proteins to the extracellular medium that denote alterations in intercellular communication. We will build on these findings to explore an overall picture of organelle homeostasis in aging and in age-related diseases.

